# Dietary Management of Eosinophilic Esophagitis in Patients on a Ketogenic Diet for Refractory Epilepsy: A Retrospective Clinician Reported Case Series

**DOI:** 10.3390/nu17233633

**Published:** 2025-11-21

**Authors:** Kelly Urbanik, Vikram Prakash, Akash Pandey, Rebecca Jennings

**Affiliations:** 1 Orlando Health Children’s Neuroscience Institute, Orlando, FL 32835, USA; vikram.prakash@orlandohealth.com; 2 Orlando Health Pediatric Center for Digestive Health & Nutrition, Orlando, FL 32806, USA; akash.pandey@orlandohealth.com; 3 Medical Affairs, Ajinomoto Cambrooke, Inc., Ayer, MA 01432, USA; rjennings@cambrooke.com

**Keywords:** ketogenic diet, eosinophilic esophagitis, refractory epilepsy, diet, medical nutrition therapy

## Abstract

Background/Objectives: Eosinophilic esophagitis (EoE) is a chronic inflammatory disorder of the esophagus that is challenging to treat. Ketogenic diets (KD) are used in patients with refractory epilepsy due to its well-documented efficacy for seizure control. As more individuals with refractory epilepsy are maintained on KD, clinicians are increasingly identifying EoE in this population. Methods: This is a retrospective clinician reported case series collected through a survey of registered dietitians who have managed a patient(s) on KD, who also had EoE, to describe what diet approaches were used. Data was collected on patient demographics, epilepsy diagnosis, feeding methods, diet prior to EoE diagnosis, endoscopy findings, EoE treatment, diet after EoE diagnosis, follow-up endoscopy results, and treatment response. Results: Nine unique cases were reported. In nearly all cases (except one), clinicians implemented dietary modifications following an EoE diagnosis alongside conventional medical therapies. The dietary strategies varied: three received an extensively hydrolyzed whey-based ketogenic formula via tube feeding, two were managed with plant-based ketogenic formulas, while one case each was placed on a complete amino acid protein plus modular ketogenic tube-fed diet, a low-dairy oral KD, and a nut-free oral KD. Only two encountered difficulties with the dietary modifications, whereas the majority reported noticeable improvements in gastrointestinal symptoms. Conclusions: This study describes several dietary approaches used to address EoE in patients following a KD. Limitations include the small and retrospective nature of the study. Further research is needed to understand the long-term efficacy and pathophysiology of these dietary interventions in those with EoE.

## 1. Introduction

EoE is a chronic, immune-mediated esophageal disorder with increasing prevalence of around 1 in 1000 [1]. Patients typically present with difficulty swallowing and food impaction. The disease displays a wide clinical spectrum, ranging from inflammatory to fibrostenotic forms, and it affects individuals across various age groups and demographics. Diagnosis is confirmed by endoscopy with histologic findings of at least 15 eosinophils per high-power field, basal layer thickening, and widened intercellular spaces, which may progress to strictures and a narrow-caliber esophagus. EoE is primarily triggered by an allergic response to dietary antigens, where epithelial signals such as thymic stromal lymphopoietin and interleukin (IL)-33 stimulate cytokines like IL-5 and IL-13, attracting eosinophils and mast cells that lead to tissue remodeling, scarring, and fibrosis. Its development is multifactorial, involving genetic susceptibility and environmental exposures [2]. Therapeutic approaches range from proton pump inhibitors and dietary modifications to topical corticosteroids and emerging biologic agents, all aiming to improve long-term outcomes. Esophageal dilation may be considered for patients with strictures and food impaction [3].

The KD is a high-fat, low-carbohydrate, and adequate protein diet therapy that is used in the dietary management of drug-resistant or refractory epilepsy. Classic ketogenic diets are usually prescribed in ketogenic ratios and are typically defined by grams of fat to combined grams of net carbohydrate (total carbohydrate minus dietary fiber) and grams of protein. Alternatively, modified Atkins diets (MAD) are designed more leniently with limited net carbohydrates and ample fats. These diets prompt the body into a state of nutritional ketosis, which occurs when the body metabolizes fat in the absence of sufficient carbohydrate [4]. KDs were first studied in patients with epilepsy in the 1920s [5] and have now been used successfully for over 100 years though its mechanism of action is still poorly understood. The anti-seizure efficacy of KDs in refractory epilepsy has been reported at 30–70% of patients responding with a greater than 50% reduction in seizure frequency and 1–33% experiencing seizure freedom [6,7,8]. These diets can be implemented orally or with tube feedings with a variety of commercially available ketogenic formulas. The medically managed KD for refractory epilepsy is highly restrictive and requires close monitoring by an experienced ketogenic team, including registered dietitians, to ensure nutritional adequacy and optimized seizure control.

As more patients with refractory epilepsy follow a restricted KD, we are increasingly encountering cases of EoE within this group similar to the general population. Alongside standard drug treatments, dietary modifications are becoming a key part of managing EoE. These dietary interventions include adopting elimination diets to limit exposure to common allergens, using specialized formulas that avoid major allergens or incorporate hydrolyzed proteins, and employing amino acid-based feeds or allergen-free home-blenderized diets. What is not clear is how these dietary interventions should be applied to those following a KD. In some instances, discontinuing the ketogenic regimen may be considered to expand food choices.

In our study, we identify the various dietary management strategies that have been used for patients with EoE who are on a KD, aiming to provide insights that can help clinicians tailor care for this complex and extremely rare patient population.

## 2. Materials and Methods

This was a retrospective clinician reported case series. An online survey was conducted via Survey Monkey^®^ (San Mateo, CA, USA) online survey tool and was directed to healthcare professionals. Surveys were sent to registered dietitians specializing in ketogenic therapy for refractory epilepsy in order to contact those who have had a patient also diagnosed with EoE. A survey invitation was sent to the International Keto Dietitians Listserv (ketodietitians Google group email listserv), which at the time the survey was conducted included approximately 490 members. Members of this listserv were practitioners, primarily registered dietitians, currently practicing in the specialty field of ketogenic diet therapy. We aimed to collect a conservative number of at least 5 cases given the presumed rarity of patients on KD who are also diagnosed with EoE. Data was collected between September and December 2024.

The survey included 11 questions and collected information from respondents about their experience in the management of patients on ketogenic therapy who also had a diagnosis of EoE.

The following data was collected:

- Patient age at the time of EoE diagnosis;
- Patient assigned sex at birth;
- Epilepsy diagnosis;
- Mode of nutrition (oral, tube-fed or both);
- Diet prior to EoE diagnosis;
- Diagnosing endoscopy findings;
- Elected treatment for EoE;
- Diet after EoE diagnosis;
- Follow-up endoscopy findings (if applicable);
- Patient outcome/response to treatment.

A subjective determination of “improved”, “stable”, or “unsatisfactory response” of EoE symptoms was made based on reported patient outcome/response to treatment description. We did not assess seizure control outcomes. All questions required a response in order to be included in the data set.

Demographic and baseline characteristics are presented in descriptive summaries and, where applicable, a mean and/or median is expressed. Counts and proportions are presented for categorical variables.

The study protocol was reviewed by the Orlando Health IRB and it was determined since subjects are healthcare professionals reflecting on their experiences with the target population that the study would be exempt status. Subjects consented by electing to participate in the survey. No protected health information was collected.

## 3. Results

**Figure 1:**
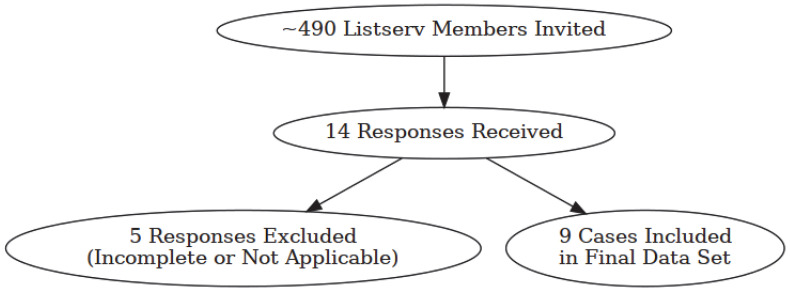
Summary of Survey Responses.

A total of 14 responses were received, and 9 reported cases were analyzed. Five survey responses were excluded, as they were incomplete (*n* = 4) or not applicable (*n* = 1). See Figure 1 for a breakdown in invitations sent to final data set. All respondents were registered dietitian/nutritionists. All completed and relevant surveys reported were included in the data analysis. These included a total of nine cases of patients with a diagnosis of EoE, confirmed by endoscopy, who were also receiving a KD. Two patients were diagnosed with EoE prior to starting the KD. All other patients (78%, *n* = 7/9) were diagnosed with EoE while already on the KD.

A summary of patient characteristics is reported in Table 1. Of the cases reported, 56% were female and 89% were children. The median age of the patients was 9 years old (ranging from 2 to 30 years). Indications for KD therapy are also included in Table 1. Patients were fed through different feeding methods with 44% being fed by feeding tube, 33% being fed by both mouth and feeding tube, and 22% being fed by mouth only.

Treatment for EoE was multifaceted, with the majority having some form of dietary management, followed by protein pump inhibitor therapy, swallowed steroids, inhaled steroids, and muscarinic agonist.

Of the KD interventions provided, the tube fed patients, including two of the patients fed by mouth and feeding tube, were transitioned to an extensively hydrolyzed whey protein-based ketogenic formula (*n* = 3), plant-based ketogenic formulas with modulars (*n* = 2), or an amino-acid based protein powder with modulars (*n* = 1). The orally fed patients, including one of the patients fed by mouth and feeding tube, were continued on an oral diet with elimination or restriction of one food, either dairy (*n* = 2) or nuts (*n* = 1). One patient was treated only with medications with no specific dietary intervention described.

All patients, except two, reported improved EoE symptom outcomes, including feeding tolerance (*n* = 5), normalized esophagogastroduodenoscopy (*n* = 2), improved clinically (*n* = 1), positive weight gain (*n* = 2), and resolution of EoE symptoms (*n* = 1). Of the two patients without reported improvement, one reported stable symptoms when continued on a classic KD by mouth and feeding tube. The other continued with episodes of reflux and inflammation on a dairy-restricted KD and protein pump inhibitor therapy. A table of individual patient descriptions are reported in Table 2. To summarize, 7/9 cases showed symptom improvement, 1/9 case reported stable symptoms and 1/9 case appeared to have worsening/unsatisfactory response. See Table 3 for a summary of case outcomes.

## 4. Discussion

This case series describes various strategies by which a KD may be implemented in a patient who is diagnosed with EoE. These included ketogenic formulas with extensively hydrolyzed whey-based protein, plant-based formulas free of most major allergens, amino acid-based modular approach, and restricted oral diets which included elimination or avoidance of dairy and nuts. All cases reported were able to either initiate or continue with ketogenic therapy for their refractory epilepsy, despite their EoE diagnosis.

The rationale for selecting one dietary approach over another in EoE remains largely speculative, as no standardized diet therapy has been universally established. However, dietary interventions commonly focus on the elimination of major food allergens, including cow’s milk, eggs, peanuts, tree nuts, soy, wheat, fish, shellfish, and sesame. For patients who are tube-fed and needing to follow a ketogenic diet, commercially available ketogenic formulas offer treatment alternatives that can exclude many of these allergens. In this study, the most frequently used formula was an extensively hydrolyzed whey-based ketogenic product. Although derived from milk, the protein in this formula is broken down into very small peptides, which may be tolerated by some individuals with milk sensitivities. Additionally, two different plant-based ketogenic formulas were utilized, both of which are free from most major food allergens. While amino acid-based ketogenic formulas are not currently available commercially, they can be custom made using individual amino acids and other nutritional modulars to create a nutritionally complete formula. One case in this study employed this approach presumably to avoid reactions to intact protein sources. For orally fed patients, dietary management typically involves the elimination of major allergens through a process of trial and error. In this cohort, individuals receiving oral nutrition had chosen to exclude dairy and/or nuts from their diets, one of whom was not successful in controlling EoE symptoms.

Another potential dietary approach to the KD for a patient diagnosed with EoE not described with this patient set is a home blenderized tube-fed diet free of major allergens. Although this approach requires more effort from families and carries an increased risk of foodborne illness, when carefully managed, it could offer another viable option for patients with EoE.

Most patients received some form of dietary intervention when diagnosed with EoE. The elected course of intervention may be best guided by what is tolerated and feasible for the patient, including a medication-only approach as was reported in one subject here. Interestingly, the two patients who continued to experience EoE symptoms were on some form of oral diet, suggesting that it may be more challenging to control allergen exposure in patients fed orally with conventional foods. Conversely, some patients on oral and tube feeding regimens were reported to have positive outcomes. When it comes to ketogenic formulas, it is not clear if there is an advantage to selecting specific protein sources to best manage the immune response that occurs with EoE. In this series, a variety of nutrition approaches were used. Most of these patients were reported to tolerate their ketogenic feeding and some of them reported a normalized EGD. Overall, the data collected is not sufficient to determine whether any one protein source offered distinct clinical advantages.

There is a need for standardized nutrition interventions for EoE, including for patients who also require KDs for the management of refractory epilepsy. One clear takeaway from this series is that a diagnosis of EoE does not necessitate the discontinuation or avoidance of ketogenic therapy. Some evidence suggests that reducing inflammation could be an important disease-modifying effect of a KD [9]. Reducing inflammation would have a beneficial effect in patients with refractory epilepsy, as well as in those patients diagnosed concurrently with EoE. In mice models, the KD, with elevated plasma ketones, showed reduced pain and inflammation thought to be related to increased levels of adenosine and/or GABA, two powerful inhibitory neurotransmitters in the nervous system [10].

Additionally, KD alters the gut microbiome, reducing microbe populations that promote pro-inflammatory cytokine signaling and increasing taxa associated with anti-inflammatory profiles [11]. This microbial shift may also influence Th2-driven pathways, which are central to EoE pathogenesis. Dysbiosis in EoE has been linked to enhanced Th2 responses and eosinophilic infiltration of the esophagus [12]. By promoting a gut environment less conducive to Th2 polarization and reducing systemic inflammatory mediators, KD may plausibly mitigate the chronic inflammation characteristic of EoE. However, this mechanistic rationale would be highly influenced by a variety of factors including age, fiber intake, pharmaceutical use, host genetics, among many others, making its overall impact challenging to predict.

This study has several limitations. First, its retrospective design introduces the potential for recall bias and limits the accuracy of the data collected. The small sample size (*n* = 9) restricts the ability to conduct statistical analyses and limits the generalizability of the findings. Additionally, follow-up EGD results were unavailable for some cases post-diet intervention, although these individuals were reported to have tolerated diet changes. The absence of long-term outcome data prevents assessment of the sustained efficacy of the interventions. Furthermore, there was considerable variability in both medical treatments and dietary approaches across cases, making it difficult to isolate the effects of any single intervention. The concurrent use of medications, such as PPIs and corticosteroids, further complicates attribution of clinical improvement solely to dietary modifications. Lastly, the sample was drawn from a specialized dietitian listserv, which may have introduced selection bias toward more successful or memorable cases, potentially skewing the results.

Further research with a larger patient pool and standardized follow-up, including evaluation of seizure control outcomes following dietary changes for EoE, is needed to validate and expand upon these findings. Capturing data on medical and dietary interventions, diet tolerance, inflammatory markers, endoscopic/histologic scores, long-term outcomes, adverse events, and impact on seizure control in this population would establish the evidence needed to clarify effective interventions and help guide practice.

## 5. Conclusions

There are different dietary strategies to approach KD implementation in those diagnosed with EoE. Our findings provide a foundation for further investigation and highlight the potential for use of KDs for the dietary management of refractory epilepsy in patients also diagnosed with EoE. These results suggest EoE is not a contraindication to a KD, but ideal nutrition intervention in this group needs further exploration.

## Figures and Tables

**Table 1:** Summary of patient characteristics from clinician surveys describing individuals following a ketogenic diet who also have eosinophilic esophagitis (EoE).

*N*	9
Age range (median), years	2–30 (9)
Female	56%
Epilepsy diagnosis	Intractable Epilepsy, (8)
	*Unspecified (4)*
	*Lennox–Gastaut (3)*
	*Aicardi Syndrome (1)*
	Glucose Transporter Type-1 Deficiency Syndrome (1)
Feeding method	Both (4)
	Tube (3)
	Oral (2)
Treatment utilized for EoE	Diet change (8)
	Proton Pump Inhibitor (7)
	Swallowed steroids (3)
	Inhaled steroids (1)
	Muscarinic agonist (1)

**Table 2:** Detailed characteristics of individuals following a ketogenic diet (KD) who also were diagnosed with eosinophilic esophagitis (EoE).

Case	Age (Year)/Gender	Feeding Method	Diet Prior to EoE Diagnosis	Elected Treatment	KD After EoE Diagnosis	Description of Reported EoE Symptom Outcomes
1	5/F	Tube fed	KetoCal^®^ 4:1 LQ (Nutricia) ^i^ + Modulars	Formula change	KetoVie^®^ 4:1 Plant-Based Protein (Ajinomoto Cambrooke, Inc.) ^iv^ + amino acids	Improved. Observed tolerated feeds. No follow-up EGD. ^b^
2	15/M	Tube fed	KetoCal^®^ 4:1 Powder (Nutricia) ^ii^	Formula change, PPI, ^a^ pancreatic enzymes	KetoVie^®^ 4:1 Peptide (Ajinomoto Cambrooke, Inc.) ^v^	Improved. Co-occurred with disaccharidase deficiency. Observed tolerated feeds. Positive weight gain. Normal follow-up EGD.
3	11/F	Tube fed	KetoCal^®^ 4:1 LQ (Nutricia) ^i^ + modulars	Formula change	Modular KD with amino acids + fats	Improved. Observed clinical improvement with fewer GI ^d^ symptoms and positive weight gain. No follow-up EGD.
4	11/F	Orally fed	CKD ^c^	Eliminated some dairy, PPI	Oral KD, low dairy	Unsatisfactory response. Reflux and episodes of inflammation continued. No follow-up EGD.
5	30/M	Orally fed	CKD	Elimination diet	Nut-free KD	Improved. Initially discontinued KD and followed elimination diet. Diagnosed nut allergy and EoE symptoms resolved with nut-free diet. Restarted nut-free KD. No follow-up EGD.
6	3/M	Both	MAD ^e^ + KetoCal^®^ 4:1 LQ (Nutricia) ^i^	Formula change, eliminated dairy, PPI, swallowed steroids, inhaled steroids	KetoVie^®^ 4:1 Peptide (Ajinomoto Cambrooke, Inc.) ^v^ + tastes by mouth.	Improved. Inlet patches seen proximal esophagus, otherwise normal follow-up EGD.
7	4/M	Both	KD	PPI, monoclonal antibody blocking interleukin-4	Oral and Tube KD—not described	Stable. No follow-up EGD.
8	2/F *	Both	Purees by mouth + Pediasure^®^ 1.5 (Abbott Nutrition) ^iii^	Formula change, dairy free, PPI	KetoVie^®^ 4:1 Peptide (Ajinomoto Cambrooke, Inc.) ^v^ + modular	Improved. Initially utilized Neocate^®^ (Nutricia) ^vii^ + dairy free purees by mouth after EoE diagnosis before transitioning to KD. Observed tolerated feeds. Follow-up EGD showed mild eosinophilia below EoE threshold.
9	9/F *	Both	Reg diet	Elimination diet, swallowed steroids, PPI, Muscarinic agonist-1	Keto Peptide (Functional Formularies^®^) ^vi^ + modulars.	Improved. Initially utilized Nourish (Functional Formularies^®^) + Real Food Blends^®^ (Nutricia) ^viii^ before transitioning to KD. Observed tolerated feeds. Follow-up EGD remained diagnostic of EoE.

* Pt not on KD at time of EoE diagnosis. (^a^) PPI: proton pump inhibitor, (^b^) EGD: esophagogastroduodenoscopy; (^c^) CKD: classic ketogenic diet; (^d^) GI: gastrointestinal; (^e^) MAD: modified Atkins diet. (^i^) KetoCal^®^ 4:1 LQ (Nutricia, Utrecht, The Netherlands): a liquid, casein dominant protein, 4:1 ketogenic formula; (^ii^) KetoCal^®^ 4:1 Powder (Nutricia): a powdered, casein dominant protein, 4:1 ketogenic formula; (^iii^) Pediasure^®^ 1.5 (Abbott Nutrition, Colombus, OH, USA): a standard liquid pediatric formula with 1.5 kcal/mL; (^iv^) KetoVie^®^ 4:1 Plant-Based Protein (Ajinomoto Cambooke, Inc., Ayer, MA, USA): a liquid, pea protein based, 4:1 ketogenic formula; (^v^) KetoVie^®^ 4:1 Peptide (Ajinomoto Cambrooke, Inc.): a liquid, extensively hydrolyzed whey-based protein, 4:1 ketogenic formula; (^vi^) Keto Peptide (Functional Formularies^®^, West Chester, OH, USA): a plant-based, 2.4:1 ketogenic formula; (^vii^) Neocate^®^ (Nutricia): a powdered, amino acid based, standard pediatric formula; (^viii^) Nourish (Functional Formularies^®^) and Real Food Blends^®^ (Nutricia): liquid whole food based, standard pediatric formulas.

**Table 3:** Diet summary and outcomes table.

Case	Feeding Method	Diet Intervention Primary Protein Source	EoE Symptom Outcomes
1	Tube fed	Pea and hydrolyzed pea protein	Improved No follow-up EGD
2	Tube fed	Extensively hydrolyzed whey protein	Improved Documented by normal EGD
3	Tube fed	Modular amino acids	Improved No follow-up EGD
4	Orally fed	Varied oral diet—low dairy	Unsatisfactory response No follow-up EGD
5	Orally fed	Varied oral diet—nut free	Improved No follow-up EGD
6	Both	Extensively hydrolyzed whey protein	Improved Documented by normal EGD
7	Both	Unknown	Stable No follow-up EGD
8	Both	Extensively hydrolyzed whey protein	Improved Follow-up EGD showed mild eosinophilia below EoE threshold
9	Both	Hydrolyzed pea protein, almond butter, coconut, buckwheat groats	Improved Follow-up EGD remained diagnostic of EoE

## Data Availability

Data set available upon request from the authors. The raw data supporting the conclusions of this article will be made available by the authors on request.

## References

[1] Dellon E.S., Hirano I. (2018). Epidemiology and Natural History of Eosinophilic Esophagitis. Gastroenterology.

[2] Khokhar D., Marella S., Idelman G., Chang J.W., Chehade M., Hogan S.P. (2022). Eosinophilic esophagitis: Immune mechanisms and therapeutic targets. Clin. Exp. Allergy.

[3] Reed C.C., Dellon E.S. (2019). Eosinophilic Esophagitis. Med. Clin. N. Am..

[4] Kossoff E.H., Zupec-Kania B.A., Auvin S., Ballaban-Gil K.R., Christina Bergqvist A.G., Blackford R., Buchhalter J.R., Caraballo R.H., Cross J.H., Dahlin M.G. (2018). Optimal clinical management of children receiving dietary therapies for epilepsy: Updated recommendations of the International Ketogenic Diet Study Group. Epilepsia Open.

[5] Wheless J.W. (2008). History of the ketogenic diet. Epilepsia.

[6] Neal E.G., Chaffe H., Schwartz R.H., Lawson M.S., Edwards N., Fitzsimmons G., Whitney A., Cross J.H. (2009). A randomized trial of classical and medium-chain triglyceride ketogenic diets in the treatment of childhood epilepsy. Epilepsia.

[7] Lin A., Turner Z., Doerrer S.C., Stanfield A., Kossoff E.H. (2017). Complications During Ketogenic Diet Initiation: Prevalence, Treatment, and Influence on Seizure Outcomes. Pediatr. Neurol..

[8] Ashrafi M.R., Hosseini S.A., Zamani G.R., Mohammadi M., Tavassoli A., Badv R.S., Heidari M., Karimi P., Malamiri R.A. (2017). The efficacy of the ketogenic diet in infants and young children with refractory epilepsies using a formula-based powder. Acta Neurol. Belg..

[9] Masino S.A., Rho J.M., Noebels J.L., Avoli M., Rogawski M.A., Olsen R.W., Delgado-Escueta A.V. (2012). Mechanisms of Ketogenic Diet Action. Jasper’s Basic Mechanisms of the Epilepsies.

[10] Ruskin D.N., Kawamura M., Masino S.A. (2009). Reduced Pain and Inflammation in Juvenile and Adult Rats Fed a Ketogenic Diet. PLoS ONE.

[11] Ang Q.Y., Alexander M., Newman J.C., Tian Y., Cai J., Upadhyay V., Turnbaugh J.A., Verdin E., Hall K.D., Leibel R.L. (2020). Ketogenic Diets Alter the Gut Microbiome Resulting in Decreased Intestinal Th17 Cells. Cell.

[12] Angerami Almeida K., de Queiroz Andrade E., Burns G., Hoedt E.C., Mattes J., Keely S., Collison A. (2022). The microbiota in eosinophilic esophagitis: A systematic review. J. Gastroenterol. Hepatol..

